# A nonsense mutation in S-antigen (p.Glu306*) causes Oguchi disease

**Published:** 2012-05-12

**Authors:** Nadia K. Waheed, Ahmed H. Qavi, Sarah N. Malik, Maleeha Maria, Moeen Riaz, Frans P. M. Cremers, Maleeha Azam, Raheel Qamar

**Affiliations:** 1 Shifa College of Medicine, Islamabad, Pakistan; 2 Shifa International Hospital, Islamabad, Pakistan; 3 Department of Biosciences, Faculty of Science, COMSATS Institute of Information Technology, Islamabad, Pakistan; 4 Department of Human Genetics, Radboud University Nijmegen Medical Centre, Nijmegen, The Netherlands; 5 Nijmegen Centre for Molecular Life Sciences, Radboud University Nijmegen, Nijmegen, The Netherlands

## Abstract

**Purpose:**

Genetic studies were performed to identify the causative mutation in a 15-year-old girl diagnosed with congenital stationary night blindness (CSNB) presenting Mizuo-Nakamura phenomenon, a typical Oguchi disease symptom. The patient also had dural sinus thrombosis (DST), thrombocytopenia, and systemic lupus erythematosus (SLE).

**Methods:**

Mutation analysis was done by sequencing two candidate genes, S-antigen (*SAG*; arrestin 1), associated with Oguchi type 1, and rhodopsin kinase (*GRK1*), associated with Oguchi type 2. In addition, the C677T variation in the methylenetetrahydrofolate reductase (*MTHFR*) gene was also screened in the family, to determine its probable association with hyperhomocysteinemia in the patient.

**Results:**

Sequencing of the *SAG* and *GRK1* resulted in identifying a novel homozygous nonsense mutation (c.916G>T; p.Glu306*) in *SAG*, which in unaffected siblings either was present in a heterozygous state or absent. The C677T heterozygous allele in the *MTHFR* gene was found to be associated with hyperhomocysteinemia in the patient and other family members.

**Conclusions:**

This is the first report of Oguchi type 1 in a Pakistani patient due to a nonsense mutation (c.916G>T; p.Glu306*) in *SAG*. The neurologic and hematological abnormalities likely are not associated with the *SAG* variant.

## Introduction

Oguchi disease is a congenitally inherited non-progressive disease of the retinal rod photoreceptor cells characterized by stationary night blindness in which patients usually possess normal visual acuity, visual field, and color vision. The distinctive attribute of the disease is the Mizuo-Nakamura phenomenon, which is characterized by a diffuse yellow or gray coloration of the retina that is absent when the rod cells are dark-adapted but reappears shortly after exposure to light [1,2]. Based on mutations in two genes, namely, the arrestin (also called S-antigen, *SAG*) and rhodopsin kinase (also called G-protein-dependent receptor kinase 1, *GRK1*) [3–6], the disease is classified as type 1 or type 2, respectively. Of these two genes, *SAG* acts as an inhibitor of the activated phototransduction cascade where the gene binds to the photoactivated-phosphorylated rhodopsin, thereby preventing the transducin-mediated activation of phosphodiesterase, thus making it a vital factor in the recovery phase of phototransduction [7].

*SAG* is expressed not only in the retina, specifically in the rod photoreceptor cell outer segment, but also in the pineal gland where the gene’s function still needs to be elucidated [8]. The SAG protein consists of 405 amino acids encoded by a gene consisting of 16 exons of which 15 are coding. To date, three nonsense mutations and a frameshift mutation (c.926delA; p.N309Tfs*12), previously annotated as c.1147delA, have been reported. The latter variant is the most frequent cause of Oguchi disease in Japanese patients [4,9–11]. The same mutation has been shown to be associated with retinitis pigmentosa [12]. In addition a mutation in *SAG* has been described previously as causing Oguchi disease in an Indian family [13]. In the Pakistani population, *GRK1* mutations have been reported to cause not only the typical form of the disease (type 2) but also its variant types [14,15]. However, to date no *SAG* mutations have been shown to cause Oguchi disease in Pakistani patients. We report here a novel homozygous nonsense mutation in *SAG* in an individual with Oguchi disease and multiple neurologic and hematological disorders.

## Methods

### Ethics committee approval and patient recruitment

The study was conducted after approval was obtained from the Shifa College of Medicine/Shifa International Hospital Ethics Committee/Institutional Review Board. The medical and family history of the patient was recorded, and blood samples were collected from the patient and family members after they had given their informed written consent for participation in the study.

### Clinical presentation and patient history

A 15-year-old girl with bilateral eye pain and headache on reading and non-progressive blurring of distance and near vision, especially in the dark, was referred by a neurologist. A detailed ophthalmological examination was performed that included visual field analysis, visual acuity testing, as well as dark adaptation and electroretinography (ERG).

In addition to Oguchi disease, the patient was diagnosed with dural sinus thrombosis (DST), thrombocytopenia, and systemic lupus erythematosus (SLE).

### Genetic analysis

**Figure 1:**
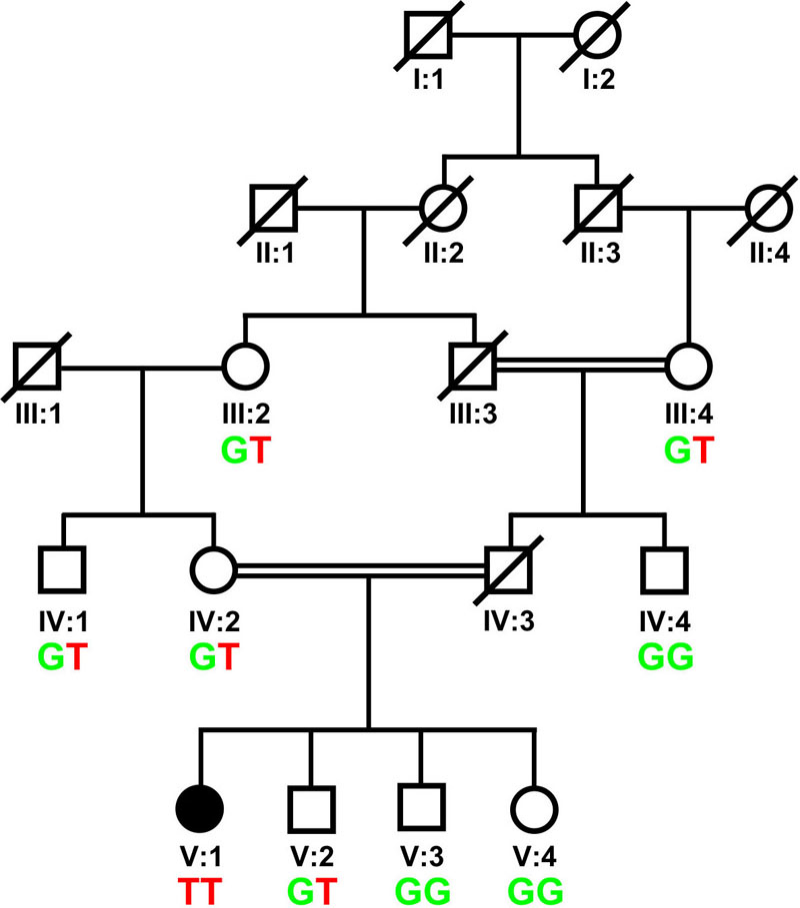
Family pedigree and mutation segregation. Pedigree of the patient and her family where the white circles represent normal women, the filled circle the affected woman, and white squares normal men. Deceased individuals are shown with a slanted line across the symbol. The segregation of mutation is also shown where the mutant nucleotide c.916T is depicted in red lettering; the wild-type c.916G in green lettering.

To identify the genetic cause of the disease in the patient, blood samples were collected by venipuncture in EDTA vacutainer tubes (Becton Dickinson, Franklin Lakes, NJ) from the patient and the available family members (Figure 1). The samples were stored at 4 °C till DNA isolation was performed as described previously [16]. Oguchi disease candidate gene analysis was performed in the family by sequencing *SAG* and *GRK1*. The latter gene was sequenced by using the primers and conditions as described previously [14,15]. To sequence *SAG* (NM_000541.4), primers flanking exons and splice sites, which included exon-intron boundaries, were designed, and the genomic DNA was amplified with PCR using the primers and conditions given in Table 1. The PCR products were purified with the GeneJET PCR purification kit (Fermentas, Glen Burnie, MD) and sequenced with the help of dye-termination chemistry (BigDye Terminator, version 3 on a 3730 or 2100 DNA analyzer; Applied Biosystems, Foster City, CA) and the same primers as used in PCR amplification. Carrier screening of the identified mutation in the family was also performed with sequence analysis of exon 11 in all family members.

**Table 1:** Sequencing primers for SAG and amplification conditions.

**Primer name**	**Forward primer (5′→3′)**	**Reverse primer (5′→3′)**	**Tm (°C)**	**Product size**
SAG-EX2	TGTCTTACCTTTCTCCAACCC	CCCTCAAAGAGTTTTGATGTTG	59	254
SAG-EX3	TATTGGCCAGGCTCAAACTC	TTGTTTCCAATCAGCCAGTG	59	381
SAG-EX4	CCTTTGCCTGACTTTTCTTTC	CCTCTGCCTTCCTGTCTCTC	59	291
SAG-EX5	CCATTCCGTCAGTGGTGG	CTATCCCCTTTCCTTTGCC	59	344
SAG-EX6	AGGCAGGAAATTTTGGGAAG	CACTTGAGCCCAGAAACCAC	55	400
SAG-EX7	ATCATGTGCCCTGTGTGAG	ACAGAGACAAGGTGGAGGTC	52	246
SAG-EX8	TGACAGTGGGGAGAGAACAG	TGAAGAGAGGGGTGTGGG	55	286
SAG-EX9	TTCCAGTGAAAGGGATTGAG	GTGACCTCTCAGGAAACAGG	52	254
SAG-EX10	GGAGAGACCAGCGTGTACC	CTTCTTCAGCAATAAACGGC	59	246
SAG-EX11	TGCCTAATGTCAAATAGGGG	TGATGTGAAGGGAAGCAGAC	59	288
SAG-EX12	CTCGAATGGAAAGGCTGC	CAGGAAAGGAAAAGTTCAGAC	55	228
SAG-EX13	TCTGAATCATGGGAAAGGG	AGAAACCGTTTTGGAGCC	59	173
SAG-EX14	GGATCTTTTGTGACTCTCCG	GAGATGCGGTCAAGAAAGAC	59	242
SAG-EX15	CATGAACTGCATGTATCTAGGC	TAAGCACTAGGGAGCAGACG	58	315
SAG-EX16	TTGATCAGTTCCTTCGTTGC	AAAGGACTAAACTGTGGGGC	58	279

Healthy control panel screening was performed with polymerase chain reaction-restriction fragment length polymorphism analysis; the presence of the mutant residue T at position c.916 in exon 11 abolishes a recognition site of BssSI. PCR was performed using the primers and conditions as described above, which resulted in the generation of a 288 bp fragment. Individuals carrying the ancestral allele upon digestion with BssSI gave two fragments (186 bp and 102 bp), while in individuals carrying the variant allele the 288 bp fragment remained undigested.

### Methylenetetrahydrofolate reductase gene analysis for hyperhomocysteinemia

A raised serum homocysteine level has been reported to be associated with C677T variation in the methylenetetrahydrofolate reductase (*MTHFR*) gene, in the homozygous as well as heterozygous state; therefore, we assessed the family members by genotyping them for this variation [17] and determining their homocysteine levels [18] to find any probable role of this single nucleotide polymorphism (SNP) in causing hyperhomocysteinemia in the patient.

## Results

**Figure 2:**
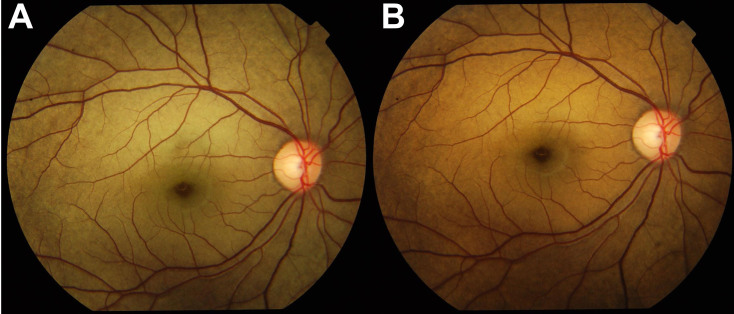
Dark adaptation test of the patient. Fundus photographs of the patient before (**A**) and after (**B**) dark adaptation. The fundus appearance was characteristic of Oguchi disease with grayish-yellow discoloration that had normal appearance after 2–3 h of dark adaptation.

Ophthalmological examination showed that the uncorrected visual acuity of the patient (V:1) was 6/9 for both eyes. Ophthalmoscopy showed static hemeralopia (which is clinically correlated to the side effect of medication) with a grayish-yellow metallic sheen of the fundus in the peripheral area (Figure 2A). The photopic ERG was normal while the scotopic ERG of the patient showed significantly reduced rod response (data not shown). After prolonged dark adaptation, the grayish-yellow fundus appearance reverted to normal (Figure 2B), which is typical in patients with Oguchi disease.

The non-ocular features, that is, neurologic symptoms in concordance with DST, included nausea, depression, and epistaxis. A brain computerized axial tomography scan showed extensive venous infarcts in the right cerebellum, pons, midbrain, right thalamus, and temporal lobe, as well as diffuse brain edema. Within this infarcted area, an interval development of hemorrhage in the right basal ganglia was also noted. The patient’s hematological reports for the SLE diagnosis revealed elevated prothrombin time (latest 16 s) and a platelet count of 137,000. Her rheumatology reports showed negative lupus anticoagulant, elevated anti-dsDNA of 83 IU/ml, and positive antinuclear antibody. Her fasting serum homocysteine was also raised 21.4 μmole/l (normal range: 4–15 μmole/l), while her protein C, glucose random, and lactate dehydrogenase levels were normal. In addition, she had malar erythema and ankle edema.

**Figure 3:**
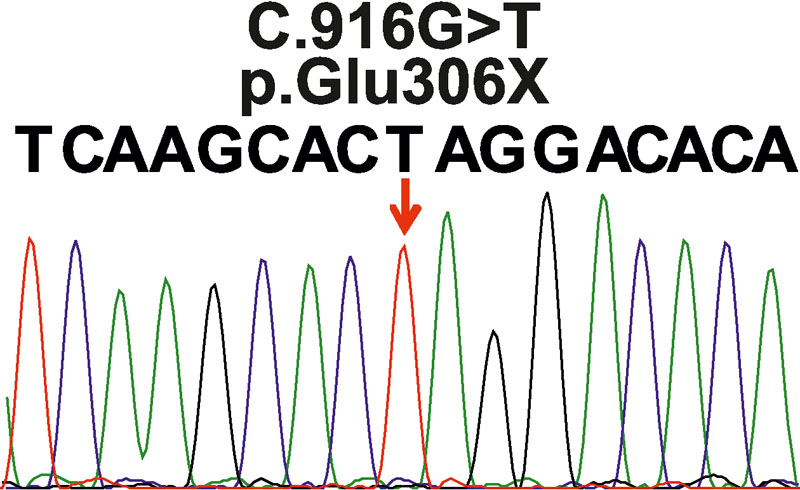
Sequence chromatogram of SAG c.916G>T variant. Sequence trace of part of exon 11 of *SAG* in the patient carrying the homozygous c.916T mutation which is indicated by a red arrow.

The patient’s *GRK1* sequence analysis did not reveal any variants, but the *SAG* screening showed a novel homozygous nonsense mutation, c.916G>T (p.Glu306*), in exon 11 (Figure 3), which was not found in a healthy control panel from the same population. Segregation analysis showed that the mutated c.916G>T allele is inherited by descent, as the patient’s (V:1) paternal and maternal grandmothers (III:4 and III:2, respectively) are first cousins and were carriers of c.916G>T (Figure 1). In addition, individuals IV:1 (maternal uncle), IV:2 (mother), and V:2 (brother) were also carriers of this mutation, while three members (IV:4 [paternal uncle], V:3 [brother], and V:4 [sister]) were homozygous for the wild-type sequence (Figure 1). The carriers of the mutation, the patient’s mother (IV:2) and brother (V:2), did not have any hematological or neurologic abnormalities.

The *MTHFR* C677T analysis showed that the patient (V:1) along with individuals IV:1, IV:2, V:2, and V:4 were heterozygous for this variation and had raised homocysteine levels that ranged between 15.90 and 22.79 µmol/l, while the wild-type homozygous CC individuals (III:4, IV:4, and V:3) had normal homocysteine levels (<15 μmol/l; Table 2).

**Table 2:** *MTHFR* (C677T) polymorphism genotypes and fasting homocysteine levels of the family members.

**Individual**	**Gender**	**Age (years)**	***MTHFR* (C677T) genotype**	**Serum fasting homocysteine (µmol/l)**
III:4	Female	70	CC	13.15
IV:1	Male	38	CT	**18.58**
IV:2	Female	40	CT	**17.76**
IV:4	Male	50	CC	14.78
V:1	Female	17	CT	**21.4**
V:2	Male	19	CT	**22.79**
V:3	Male	15	CC	10.50
V:4	Female	12	CT	**15.90**

The values in bold of homocysteine levels are greater than the normal range.

## Discussion

SAG belongs to the arrestin protein family, which are soluble cytoplasmic proteins with four identified members (arrestin 1–4). Based on differences in their structure and function, these four proteins are distributed into two sub-classes: the visual and non-visual proteins. The visual proteins, arrestin 1 and 4, are expressed specifically in the retina, while arrestin 2 and 3 are ubiquitously expressed [19,20]. Among the visual sub-family, arrestin 1 is rod specific, which is also known as S-antigen (SAG) while arrestin 4 is cone specific and called X arrestin. These visual arrestins are located in the rod or cone outer segment where they bind to their receptors (phosphorylated rhodopsin/opsin proteins) to switch off the rhodopsin/opsin-activated phototransduction cascade [21]. Arrestin knockout mice experience photoreceptor cell degeneration due to continuous upregulation of the phototransduction, as a result of the defective rhodopsin/opsin shut-off mechanism [22]. This defective mechanism results in Oguchi disease, a rare form of autosomal recessive congenital stationary night blindness caused by mutations in the genes coding for either SAG or GRK1, as both proteins are involved in terminating the activation of the phototransduction cascade, where SAG plays an important regulatory role while GRK1 functions as the phosphorylating enzyme [23,24].

**Figure 4:**
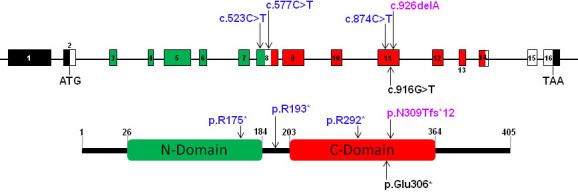
Mutation spectrum of *SAG*. The location of the known and new sequence variants are depicted above and below the gene (in cDNA notation; upper panel) and protein (in amino acid notation; lower panel), respectively. Oguchi associated mutations are indicated in blue letters while the Oguchi and retinitis pigmentosa associated mutation is shown in dark orchid color. The novel nonsense mutation (c.916G>T; p.Glu306X) is depicted in black letters below the gene and the predicted protein, which hypothetically would result in a protein with a severely truncated C domain.

Oguchi disease has been shown to be the most frequent cause of congenital stationary night blindness in the Japanese population, where the disease is frequently caused by only a single base pair deletion (c.926delA; p.N309Tfs*12) in *SAG* [4]. Apart from an association with Oguchi disease and retinitis pigmentosa (RP), the heterozygous 926delA has recently been shown to be involved in the manifestation of variable clinical phenotype in a patient without stationary night blindness [10]. In addition, though Sippel et al. [25] observed several amino acid variants in SAG in patients with RP, none of these segregated with the disease in the respective families. Researchers have also observed that mutations in *SAG* lead to the onset of RP in Oguchi patients at an advanced stage of the disease, thus resulting in overlapping phenotypes [26]. Figure 4 depicts the complete mutation spectrum of *SAG*, apart from a frameshift mutation; all previously identified variants are nonsense mutations. To date, in the Pakistani population there have been no reports of *SAG* mutations causing Oguchi disease. In the present study, we have identified a novel *SAG* variant (c.916G>T; p.Glu306*) as the cause of Oguchi disease in a female patient who inherited the mutation from a common ancestor of her parents. The identified mutation is located in a conserved motif of the C-domain of the protein (Figure 4) specifically involved in receptor binding. The position of this nonsense mutation in exon 11 suggests the resulting mRNA could be subject to nonsense-mediated decay [27,28]. If a truncated protein is synthesized, the terminal portion of the C-domain that normally spans amino acids 203 to 364 would be absent; thus, this mutant protein would not be able to activate G proteins in the visual cascade.

As the affected individual in the current study has not only Oguchi disease but also DST, thrombocytopenia, and SLE, a detailed literature search was performed to ascertain the correlation among ophthalmological, neurologic, and hematological features. SLE is a multisystem autoimmune connective tissue disorder with various clinical presentations including thrombocytopenia, a condition caused by autoimmune platelet destruction, which is present in one third of SLE cases [29]. DST represents blood clot formation inside any of the dural sinuses in the brain. The co-occurrence of DST with SLE has been seen in patients with DST-induced benign intracranial hypertension syndrome in SLE patients [30,31]. In addition, DST has been shown to be aggravated due to heparin-induced thrombocytopenia as well as thrombosis [32]. Therefore, there might be a contribution of common genetic or environmental factors in the manifestation of these three conditions (SLE, DST, and thrombocytopenia) as they are all autoimmune-related disorders. One interesting clinical aspect in this patient is hyperhomocysteinemia, which points toward the probable involvement of this factor in combination with other unknown factors in causing the onset of non-ocular features in this patient. Several studies have reported the association of hyperhomocysteinemia with autoimmune diseases such as SLE due to unknown factors [33]. In addition, other studies have shown the association of C677T polymorphism in *MTHFR* with raised serum homocysteine levels [18,34,35]. The family members C677T genotyping revealed an association of this SNP with hyperhomocysteinemia in the family; in addition, the heterozygous (CT) individuals had raised homocysteine levels compared to the homozygous (CC) family members. Several case-control association studies have shown that SLE patients with hyperhomocysteinemia are susceptible to developing cardiovascular disease (CVD) later in life [33]; in addition, the age of onset of CVD in the South Asian population is above 50 years of age [36]. As the patient and other family members with hyperhomocysteinemia are currently young, they might develop CVD later in life. We must point out here that the genetic as well as biochemical investigations into the onset of non-ocular findings in this patient still need further elucidation. As SAG is expressed specifically in the rod cells and pineal gland, in the absence of other reported Oguchi cases with DST, SLE, and thrombocytopenia, it is assumed that the non-ocular features are not associated with the *SAG* variant.

In conclusion, we have identified the first nonsense mutation in the *SAG* gene in a patient with Oguchi type 1 disease in Pakistan.
